# Effect of Ginkgo Biloba Powder on the Physicochemical Properties and Quality Characteristics of Wheat Dough and Fresh Wet Noodles

**DOI:** 10.3390/foods11050698

**Published:** 2022-02-26

**Authors:** Liangyi Li, Wenhua Zhou, Anqi Wu, Xin Qian, Le Xie, Xiaojie Zhou, Lin Zhang

**Affiliations:** 1 Hunan Key Laboratory of Processed Food for Special Medical Purpose, Changsha 410004, China; liliangyi0088@126.com (L.L.); t20041493@csuft.edu.cn (W.Z.); waq527527@126.com (A.W.); 18736993693@163.com (X.Q.); xiele4949@163.com (L.X.); xiaojiezhou2020@163.com (X.Z.); 2 National Engineering Research Center of Rice and Byproduct Deep Processing, Changsha 410004, China; 3 College of Food Science and Engineering, Central South University of Forestry and Technology, Changsha 410004, China

**Keywords:** ginkgo biloba, fresh wet noodles, rheological properties, textural properties, microstructure

## Abstract

Effects of ginkgo biloba powder (GBP) on the chemical, physicochemical properties and quality of dough and fresh wet noodles were investigated. Lower contents of gluten and starch, and higher contents of fibre, amylose and flavonoids in GBP than wheat flour, were detected. Water absorption of dough increased and the development time and stability time of dough were decreased with GBP addition. Meanwhile, the pasting properties results showed that the addition of GBP reduced the aging degree of starch and improved the thermal stability of dough. Scanning electron microscopy results showed that addition of GBP smoothed the surface of raw noodles while increasing the hole size of the cooked noodles. With increased GBP addition (0~40%), the chewiness and extensibility of the fresh wet noodles increased significantly (*p* < 0.05), and the sensory scores changed, ascending from 0~20% substitution, and then descending from 20~40% substitution. The digestibility and estimated glycemic index (eGI) values of the GBP fresh wet noodles decreased significantly (*p* < 0.05). In general, 20% GBP addition could improve the chewiness, extensibility, taste and nutrition of fresh wet noodles, and decrease the digestibility and eGI values of noodles. Thus, GBP has potential for application in the noodle industry.

## 1. Introduction

Noodles are one of the most important staple foods around the world, especially in Asian countries. Fresh wet noodles are produced without the drying process and result in a higher water content than dry noodles. Traditionally, wheat flour has been used as the main raw material for noodle preparation. Recently, to enrich the flavour, nutrition, and character of noodles, other staple foods, including vegetables, fruits, mushrooms and plants, such as oats [1], buckwheat [2], sweet potato [3] *Hericium erinaceus* [4], pitaya peel [5] and so on, have been mixed with wheat flour to produce noodles. The addition of potato flour improved the pasting characteristics of the dough and the water absorption, hardness, and elasticity of the noodles, but destroyed the gluten network structure of the dough and reduced the texture of the noodles [6]. Shiitake mushroom powder and dragon fruit peel powder could improve the color, total polyphenol content, and antioxidant activity of the noodles [4,5]. The addition of buckwheat can improve the quality of noodles, and also increase the total phenol content, total flavonoid content and antioxidant capacity of the noodles [2]. Addition of oats can increase the nutritional value of the noodles, but can also change the texture and sensory characteristics of the noodles, resulting in a decrease in product quality [7].

*Ginkgo biloba* is one of the oldest known plant species on Earth, and is known as a “living fossil of ancient life” [8]. Ginkgo biloba has been used for thousands of years in China as a traditional Chinese medicine and food. It is rich in protein, starch, polysaccharides, vitamin C, trace elements [9] and flavonoids, which have anti-inflammatory [10], antioxidant [11], and anticancer effects [12]. Up to now, ginkgo extracts were used as hypotensive drugs and cerebrovascular drugs. Further, it is also applied in foods, such as ginkgo beverages [13], ginkgo steamed eggs and ginkgo stewed chicken [14]. Ginkgo biloba contains some toxic compounds. Amygdalin-derived hydrocyanic acid and ginkgolic acid are the two important toxic species. Hydrocyanic acid can cause acute poisoning or central nervous system syndrome in humans [15], and ginkgolic acid can cause allergies and other adverse reactions [16]. Studies showed that the hydrocyanic acid content of ginkgo nuts could be reduced by 72.40% when steamed at 100 °C for 10 min [17]. The G.-J. Fan group steamed ginkgo nuts at 115 °C for 75 min, which resulted in a 54.5% reduction in ginkgolic acid content [18]. The decrease of ginkgolic acid by heat treatment of ginkgo nuts is accompanied by a reduction of hydrocyanic acid [17,19]. Therefore, ginkgolic acid concentration is commonly used as a marker to measure the content of toxic substances in ginkgo by many studies [16,17,18,19]. Steamed ginkgo nuts, which have the low content of toxic compounds and abundant nutritive compounds, could be used as raw materials for food processing.

To our knowledge, the study of fresh wet noodles processed with ginkgo biloba powder (GBP) and wheat powder is limited. Thus, the effect of GBP on the rheological properties (farinograph, pasting and dynamic rheological properties) of wheat dough, the quality characteristics (cooking and texture properties), sensory properties, and microstructure of fresh wet noodles were investigated in this study. This study will provide essential information on the nutritional value of noodles and the development of noodles produced from the mixture of wheat flour and GBP.

## 2. Materials and Methods

### 2.1. Materials and Chemicals

Wheat flour was provided by Hunan Kaixue Co., Ltd. (Changsha, China). Ginkgo nuts were purchased from Xuzhou Hui Er-kang Food Co., Ltd. (Changsha, China). The standard methods of the Association of Official Analytical Chemists (AOAC) were used to measure the protein (AOAC Method 220), moisture (AOAC Method 361) and wet gluten contents (AOAC Method 211) of ginkgo biloba powder (GBP) and for wheat flour content determination [20]. Kits for the determination of total sugar content, starch content, amylose content, glucose content, fibre content and flavonoid content were purchased from Beijing Solaibao Technology Co., Ltd. (Beijing, China). Ginkgolic acid standard, Alpha-amylase (10080, from hog pancreas, 50 U/mg), amyloglucosidase (10115, from *Aspergillus niger*, 70 U/mg), pepsin (P7000, from porcine gastric mucosa, 250 U/mg) and trypsin (T0303, from porcine pancreas, type IX-S, 18,000 BAEE units/mg protein) were purchased from Hunan Guolunmei Biotechnology Co., Ltd. (Changsha, China). Other reagents (absolute ethanol, concentrated sulfuric acid, methanol, petroleum ether, acetic acid, sodium acetate, sodium hydroxide, potassium chloride, hydrochloric acid) used in the analytical procedures were from China National Pharmaceutical Group Corporation (Wuxi, Jiangsu, China).

### 2.2. Preparation of GBP

Fresh ginkgo nuts were opened with a hammer to remove the hard shell, flesh skin, and fruit core of the nut. Ginkgo pulp was then steamed in a steamer for 35 min to reduce the ginkgolic acid content [19]. Then, the ginkgo nut and distilled water were beaten in a wall breaker at a mass ratio of 1:2 and poured into an iron plate. After being frozen in a refrigerator for 24 h, the samples were placed in a freeze dryer for vacuum drying for 48 h (B01-10NA, Ningbo Xinzhi Biotechnology Co., Ltd., Ningbo, China). Dried ginkgo nuts were crushed with a grinder (Midea MJ-PB12Easy219, Guangdong Mei’s Life Electrical Appliances Manufacturing Co. Ltd. Guangdong, China) into particles which could pass through a screen with 0.125 mm diameter. The GBP were placed in aluminium-lined bags for storage at 4 °C. All the GBP was prepared in the same conditions to ensure the accuracy of the experiment.

### 2.3. Sample Preparation

Samples of 0%, 10%, 20%, 30%, 40%, and 100% GBP substituted per 500 g of wheat flour were prepared. The GBP/wheat flour mixture samples were packed in aluminium-lined bags and stored at 4 °C.

### 2.4. Physicochemical Characterization of Samples

#### 2.4.1. Determination of Ginkgolic Acid Content

Ginkgolic acid content in GBP/wheat flour mixed powder was analyzed according to Fan et al. [18] by high performance liquid chromatography (LC-30A, Shimadzu, Kyoto, Japan). Dried powder (0.5 g) was incubated with 10.0 mL of ethanol/water (85:15, *v/v*) for 12 h, followed filtration by vacuum filtration and vaporised ethanol with a rotary evaporation. The extractum was extracted with petroleum ether, vaporised and dissolved with methanol (10.0 mL). The chromatographic column was TC-C18. The detecting wavelength was 210 nm. The mobile phase was acetonitrile (0.050%, *v/v*) mixed with trifluoroacetic acid (85:15, *v/v*). The elution speed was 1.0 mL/min. The temperature of the column was 35 °C. The standard stock was diluted in methanol into a series of lower-concentration solutions.

#### 2.4.2. Farinogram Properties

The farinograph properties were determined with a farinograph (Micro-dough LAB, Sweden porton corp, Stockholm, Sweden). Wheat flour (100 g) was replaced with different content of GBP (0%, 10%, 20%, 30%, and 40%). A certain amount of water was added into the mixer and each dough was stirred in the mixer until the consistency of 500 ± 10 FU was achieved. In order to evaluate the quality of dough. The water absorption, dough development time, dough stability time and mixing tolerance index were measured.

#### 2.4.3. Pasting Properties

The pasting properties of GBP/wheat flour mixtures were determined by a Rapid Visco Analyzer (RVA-Super 4 type; Sweden porton corp, Stockholm, Sweden) [21]. Samples (3.5 g, 14% mb) and distilled water (25 mL) were mixed to form a slurry. The tests were conducted with a programmed heating and cooling cycle for 13 min. The peak viscosity (PV), trough viscosity (TV), breakdown viscosity (BV), final viscosity (FV), setback viscosity (SV), peak time (PT), and pasting temperature (PTemp) were determined.

#### 2.4.4. Dynamic Rheological Properties

The dynamic rheological properties of the dough samples with different additions of GBP (0%, 10%, 20%, 30%, 40%) were performed with a rheometer (AR 2000ex, TA instruments, New Castle, DE, USA). According to the pre-experiments for the linear viscoelastic interval, a strain at 0.1% was chosen to perform the angular frequency sweep (0.1 to 100 rad/s). The frequency sweep was performed at 25 °C with a stainless steel plate (diameter = 40 mm) and a gap (1 mm) between plates. An equilibration time of 10 min was applied to all samples before the measurement was conducted [22]. The power-law model was employed for the data fits:

G′ = K′ × ω^n′^ (1)

G″ = K″ × ω^n″^ (2)

where: G′ is storage modulus (Pa), G″ is loss modulus (Pa), ω is angular frequency (rad/s), K′, K″ (Pa·s^n′^), n′ and n″ are experimental constants [23].

### 2.5. Preparation of Ginkgo Biloba Fresh Wet Noodles (GBN)

Fresh wet noodles were prepared using the method reported by Guan et al. [21] with minor modifications. Firstly, GBP/wheat flour (100 g) and deionised water (30 g) were mixed in a pin kneader (B5A, frequency control mixer, Guangzhou Wilbo Hotel Equipment Co., Guangzhou, China) for 10 min. The prepared dough was put into a self-sealing bag and placed in an intelligent artificial climate incubator (35 °C) and allowed to stand for 30 min (PDR-150 A, Ningbo Xinzhi Biotechnology Co., Ltd., Ningbo, China). The prepared dough was rolled with a noodle maker (DHH-180 A, Yongkang Haiou Electric Appliance Co., Ltd., Jinhua, China) five times to produce a dough piece (1 mm). The dough piece was cut into strips (width 2 mm, length 200 mm) to obtain noodles. The prepared noodles were placed in distilled water (400 mL) at room temperature (RT) (26 °C) for 1 min. Noodles were well prepared for determination. To determine the maximum addition of GBP to wheat flour at this moisture addition level, we performed a pre-experiment. When the content of GBP in the GBP/wheat flour mixture reached 42.5%, the dough could not be formed, and could not be made into noodles.

### 2.6. Cooking Properties

Cooking qualities of ginkgo fresh wet noodles were determined with the method reported by Yu et al. [24] and Zou et al. [25] with minor modifications. GBN (20 pieces) were placed into boiling water (1000 mL) and cooked. The optimum cooking time of noodles was determined by observing a white core in the center of the noodles. Samples were taken out every 30 s during cooking, and cut into 1 cm short strips and squeezed with a transparent glass plate. The time when the white core disappeared was recorded as the optimum cooking time.

A 10 g GBN sample (20 cm in length) was placed into 400 mL of boiling water until the optimum cooking time. The well-cooked GBN were drained with filter paper, and weighed with microbalance. Water absorption was calculated as the percentage increase in the weight of the cooked noodle compared to the weight of uncooked noodles. Noodle soup was collected in a volumetric flask and diluted to 400 mL with distilled water. Then 100 mL solution was taken into an aluminium box and dried to a constant weight. Cooking loss was calculated as a percentage of dry matter lost during cooking to uncooked GBN weight.

### 2.7. Texture Properties

Texture profile analysis (TPA) of cooked GBN was performed using a TA-XT plus texture analyser (TA-XT2i, Stable Micro System Ltd., Haslemere, UK) with the method described by Kang et al. [26], with some modifications. The well-cooked noodles were placed on a flat metal plate and measured with a cylindrical probe (P/36R, 36 mm). Texture profile analysis (TPA) was set to a pretest speed of 2.0 mm/s, test speed of 0.8 mm/s, post-test speed of 2.0 mm/s, strain of 70%, interval time of 1 s. The mean value of six replications was used.

Tensile strength analysis was performed with another probe Code A/SPR. Parameters were set as pretest speed of 2.0 mm/s, test speed of 2.0 mm/s, post-test speed of 10.0 mm/s, and distance of 80 mm. The tensile strength and distance were detected. The tensile strength was calculated with the equation as:

L = F/S,(3)

F was the force and S was the cross-sectional area of the noodle.

S was calculated as:

S = L × h(4)

L was width of the noodle and h was thickness of the noodle. The mean value of six replications was used.

### 2.8. Sensory Evaluation

Sensory evaluation of cooked GBN were studied by a method described by Wang et al. [27] with minor modifications. Sensory evaluations were performed under artificial daylight in sensory booths. Ten trained panel members (5 males and 5 females, 20–40 years old) from the School of Food Science and Technology were selected to perform the sensory tests. Sensory panel members were trained with the ISO standards for sensory analysis (ISO 8586:2012) and pretested with the method described in ISO 11132: 2021. The GBNs were cooked until the optimal cooking time and 10 g cooked noodles were tasted by each sensory panel member. The evaluation finished within 20 min and the mouths of sensory panel members should be rinsed with spring water between samples. The sensory evaluation standard of GBN is shown in Table 1.

### 2.9. Scanning Electron Microscope (SEM) of GBN

To investigate the surface microstructure, freeze-dried GBN was placed on specimen stubs with the surface side face up and were sprayed gold with a magnetron ion sputter metal coating device (SC7620, Quorum Company, Britain). Samples were observed at 500 and 1000 times magnification at 5.0 kV with a SEM (Zeiss Sigma300, Carl Zeiss AG, Oberkochen, Baden-Wuberg, Germany).

### 2.10. In Vitro Starch Digestibility and Estimated Glycemic Index (eGI) Value

In vitro starch digestibility of noodles was determined with the method reported by Liu et al. [28]. The glucose content was determined by glucose assay kit. The degree of starch digestion was obtained by multiplying the glucose concentration by a factor of 0.9 and then by 100. The rate of starch digestion was expressed as the percentage of total starch (TS) hydrolysed at different times.

A non-linear model established by Gori et al. [29] was applied to describe the kinetics of starch hydrolysis [30].

C_t_ = C_∞_ (1 − 2.71828^−kt^) (5)

C_t_ is the starch hydrolysis rate at time t (min), where C_∞_ is the equilibrium concentration of starch hydrolysis at the final time, k (min^−1^) is the first-order kinetic coefficient and t (min) is the digestion time.

The area under the hydrolysis curve (AUC) was calculated with the first order equation:

AUC = C_∞_(t_f_ − t_0_) − (C_∞_/k)[1 − exp^−k(tf−t0)^] (6)

t_f_ corresponds to the final time (180 min), t_0_ is the initial time (0 min). The hydrolysis index (HI) was obtained by dividing the area under the hydrolysis curve of each sample by the corresponding area of a reference sample (white bread).

The eGI was calculated using the equation reported by Granfeldt et al. [30].

eGI = 0.862 × HI + 8.198(7)

### 2.11. Statistical Analysis

All experiments were performed in triplicate and the data were reported as mean value ± standard deviation (SD). Statistical analysis (ANOVA) of the result were conducted using the student *t*-test (*p* < 0.05) using 19.0 SPSS software (IBM, Armonk, NY, USA).

## 3. Results and Discussion

### 3.1. Chemical Characterization of Wheat Flour Supplemented with GBP

The main chemical compositions of wheat flour, *Ginkgo biloba* powder (GBP), and the GBP/wheat flour mixture were detected (Table 2). The contents of moisture, protein, total sugar, starch, and gluten decreased gradually with the increase of GBP concentration, whereas the contents of flavonoid, fibre, amylose and ginkgolic acid increased. This is because pure GBP has no gluten and has lower contents of moisture (10.20%), protein (13.75 g/100 g), total sugar (639.23 mg/g) and starch (34.83 g/100 g) than pure wheat flour. Due to the high concentration of total flavonoids (2.00 mg/g) in GBP, the contents of flavonoids were increased in the GBP/wheat flour mixtures. Ginkgolic acid is one of the toxic compounds in GBP. After the pretreatment process described in Section 2.2, the content of ginkgolic acid in the pure GBP and the mixed powder with 40% GBP addition was 2.06 mg/kg and 0.82 mg/kg, respectively. The content of ginkgolic acid in other mixed powders was even lower. According to the European Pharmacopoeia, Chinese pharmacopoeia and studies on the ginkgolic acid toxicity, the safe dosage of ginkgolic acid should be below 100 mg/kg [31,32,33]. Therefore, it is safe to add GBP to wheat flour. Meanwhile, the addition of GBP can increase the flavonoid content and improve the nutritional properties of the mixed powder, but the lower contents of water, protein, starch, and gluten may effect farinogram properties, pasting properties, and dynamic rheological properties of dough, and then affect the quality of ginkgo biloba noodles (GBN).

### 3.2. Farinograph Properties

Farinograph properties are one of the important characterization parameters of dough, and can indicate the kneading resistance and the processing performance. As shown in Table 3, when the concentration of GBP increased from 0% to 40%, water absorption was increased. Similar changes were observed by Sudha and co-workers [34], on blending bran with wheat flour. This may due to the higher fibre content in GBP. The hydroxyl group in fibre can combine with more water through hydrogen-bond interaction and enhance water absorption [34,35].

When the GBP content increased from 0% to 40%, dough development time and mixing tolerance index of dough increased and the dough stability time decreased. This is due to the higher content of fibre and lower content of gluten in GBP/wheat flour mixtures (Table 2). The fibre which has hydroxyl groups can compete the water absorption with gluten, which results in the longer time for gluten network formation [34,36]. Meanwhile, the dough with the lower gluten content also takes a longer time to form the gluten network structure [37,38,39]. Thus, the longer gluten network formation time resulted in a longer dough development time. Meanwhile, the lower gluten content decreased the stability of the gluten network and resulted in the higher tolerance index of dough and shorter dough stability time [40].

### 3.3. Pasting Properties

Pasting properties can mainly reflect the expansion capacity of starch and binding water capacity of starch and water in the compound flour system. In addition, it can also reflect the influence of starch pasting properties on the edible quality of pasta products. As shown in Table 4, when the concentration of GBP in the mixture increased from 0% to 40%, the peak viscosity of the dough decreased from 2621.00 cp to 1268.67 cp, the trough viscosity decreased from 1943.33 cp to 894.67 cp, and the final viscosity decreased from 3091.67 cp to 1693.33 cp. These results indicate that the addition of GBP would reduce the viscosity of the mixed flour. This is possibly due to the high content of amylose, and similar phenomena were reported by other studies [41,42].

Breakdown value (BD = PV − TV) is related to the thermal stability of starch paste at high temperatures and the stability during shearing. When the GBP concentration increased from 0% to 40%, the breakdown values decreased from 677.67 cp to 374.00 cp. These results indicate that the addition of GBP could increase the thermal stability and anti-shear property of the mixed flour. These changes may due to the higher fibre and amylose concentrations in GBP [35].

The setback value is related to the recrystallization of starch molecules in the starch pastes [43]. When the GBP concentration increased from 0% to 40%, the setback value decreased from 1148.33 cp to 798.67 cp. These results indicate that the GBP addition could decrease the recrystallization level of starch molecules and then inhibit the retrogradation of the starch. This may due to the lower starch concentration in GBP than in pure wheat flour powder.

### 3.4. Dynamic Rheological Properties

**Figure 1:**
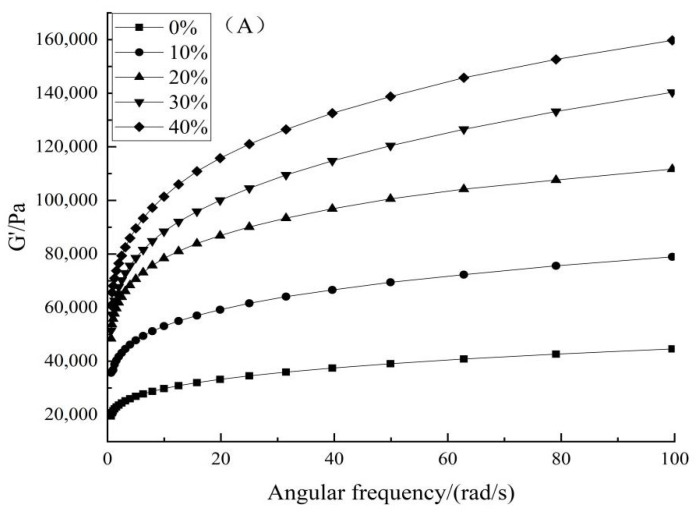

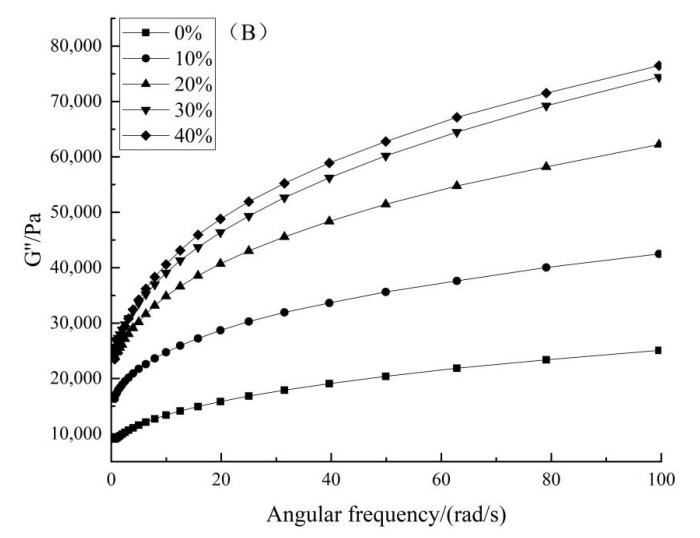
Effects of wheat flour supplemented with different contents of GBP on dynamic rheological properties of dough. Storage moduli (G′) (**A**) and loss moduli (G″) (**B**).

The effects of GBP addition on the dynamic rheological properties of dough were obtained by measuring viscoelasticity and viscosity (Figure 1). The storage modulus (G′) (Figure 1A) and loss modulus (G″) (Figure 1B) of the dough as a function of frequency were detected, and describe the elasticity and viscosity of the dough. In the angular frequency range of 0.01–100 rad/s, both G′ and G″ values of the mixed dough increased with the content of GBP, and G′ of the dough was higher than G″ (Figure 1A,B). This indicates that the dough mainly exhibits elastic behavior. This is because fibres in GBP contain a large number of hydroxyl groups that can bind more tightly to water molecules, which changed the water distribution within the dough, and increased the viscoelasticity of the dough [44,45]. In addition, fibre is a component with high swelling capacity, and the addtion of the higher fibre content GBP would also enhance the elasticity of the dough [46].

Power-law equations were employed for fitting the G′ and G″ values. As shown in Table 5, the dependence of the dynamic moduli of G′ and G″ on the angular frequency can be well-fitted by Equations (1) and (2) for an angular frequency range from 1 to 100 rad/s. The values of the coefficients of determination (R^2^) for G′ and G″ are greater than 0.996 and 0.977, respectively. It indicates that the power-law model can well describe the viscoelastic properties of GBP dough samples. The values of K′ are higher than the values of K″, and the values of n’ are lower than those of n″. The K′ values increased with the increase of GBP contents. When the content of GBP increased from 0% to 30%, the K″ values increased with the increase of the GBP contents. When the content of GBP reached to 40%, the K″ value was lower than that of 30% GBP content dough. Meanwhile, GBP contents have a slight effect on the n′ and n″ values. These results indicate the GBP could enhance the viscoelastic properties of the wheat flour dough. However, at the high GBP content (40%) this influence would be decreased. Similar results were obtained in Korus’ work [46]. This is possibly due to the high fibre and amylose contents.

### 3.5. Cooking Properties

Cooking time, water absorption and cooking loss were employed for evaluating the cooking quality of GBN. As shown in Table 6, when the GBP concentration increased from 0% to 40%, the optimal cooking time of GBN increased from 336.66 s to 433.33 s. The water absorption of the noodles increased from 4.10% to 11.20%. These results were probably due to the high fibre content of GBP. Fibre has a high water-absorption capacity, which could increase the water absorption of GBN. Similar changes were observed by Zhongfu Cao and co-workers [47] when blending soy protein isolate with wheat flour.

Cooking loss not only reflects the solid content lost to cooking water during the cooking time, but also represents chimerism between starch and protein, and the integrity of the noodle network structure [24]. As shown in Table 6, when the GBP concentration was increased from 0% to 40%, the cooking loss increased gradually, rising from 4.10% to 11.20%. A similar phenomenon was obtained by Chunsheng Tao and co-workers, who found the addition of exogenous starch to noodles could significantly increase the cooking loss of noodles [48]. This may be highly related to the fact that the addition of GBP reduces the gluten concentration in the dough and the stability of the gluten–protein network in noodles. Furthermore, starch particles can’t be wrapped tightly, leading to the increase of starch dissolution and cooking loss during the cooking time.

### 3.6. Texture and Tension Properties

Hardness, adhesiveness, springiness and chewiness of noodles are the important parameters of noodles texture characters, and highly related with the taste quality of noodles [47]. As shown in Table 7, as the concentration of GBP increased from 0% to 40%, the corresponding hardness increased from 2588.13 g to 3301.09 g (*p* < 0.05), adhesiveness increased from 64.73 g·s to 125.29 g·s., springiness increased from 88.01 g·s to 92.44 g·s, and chewiness increased from 1499.79 g·s to 1981.41 g·s. The hardness and chewiness of GBN increased with GBP addition. This may be due to the higher fibril concentration of GBP, which increased the protein–polysaccharide (fibre) gel system through crosslinking action in the GBN. Adhesiveness is related to the dissolution rate of starch and the adhesion of the noodle’s surface [49]. The increase of adhesiveness may due to the lower gluten concentration of GBP, which reduced the stability of the gluten network in noodles. Furthermore, the higher concentration of amylose of GBP would also increase the dissolution of amylose during the cooking process of noodles and cause increased viscosity of the noodles. The higher springiness may due to the higher concentration of fibril and the starch gelatinization [48].

The tensile strength and elasticity distance are two important properties of noodles [50]. The elasticity strength relates the toughness of the noodles, and the tensile distance represents the ductility of noodles. With the addition of GBP (up to 40%), the tensile strength increased from 17.37 g to 32.26 g, and the tensile distance extended from 24.38 mm to 35.08 mm. These results indicated that the addition of GBP had a strengthening effect on the ductility and elasticity of noodles. Thus, the addition of GBP improved the texture and tension characteristics of noodles.

### 3.7. Sensory Properties

Snsory properties, including color, appearance, palatability, toughness, viscosity smoothness and flavor, are the important properties of noodles. As shown in Table 8, when the GBP concentration was at the range of 0% to 20%, the sensory indexes such as color, appearance, toughness, viscoelasticity and flavor of noodles increased with the rise of GBP concentration, and when the concentration of GBP was at 20%, the scores of these indexes were reached to the maximum values. The palatability and smoothness reached the maximum values when the GBP addition was at 10%. Flavor has the maximum value at 30% GBP addition. When the GBP content exceeded 20%, the sensory quality of GBN decreased gradually. This may be because when there is high GBP addiction, there is a much lower concentration of gluten, and the higher flavone content reduced the sensory properties of noodles.

### 3.8. Microstructure

**Figure 2:**
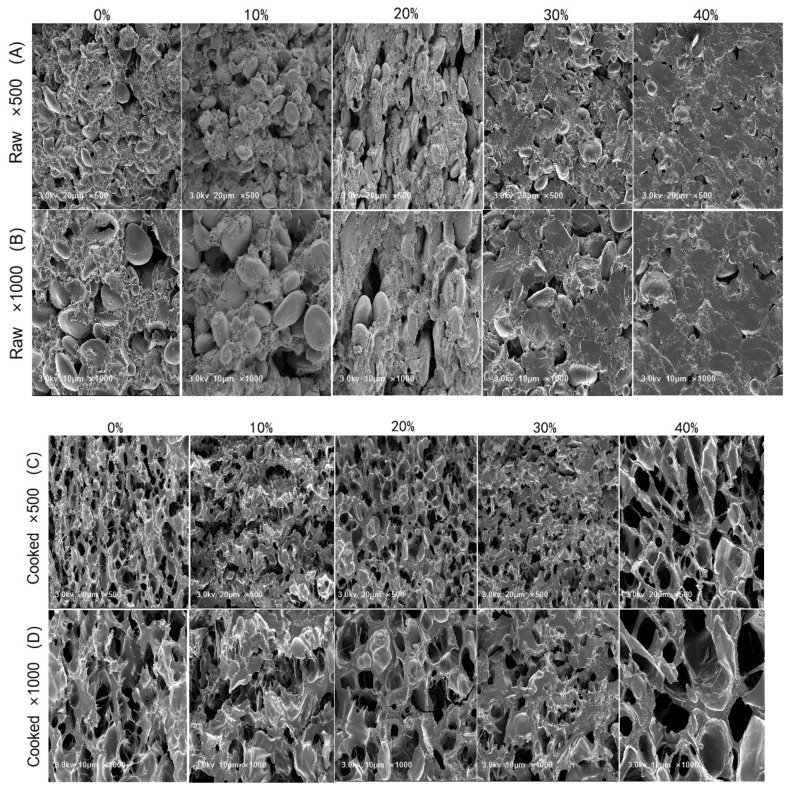
Scanning electron micrographs of noodles substituted with different level of GBP.

The microstructure of raw GBN and cooked GBN with different GBP addition levels was detected by scanning electron microscopy (SEM). As shown in Figure 2, when the GBP addition increased from 0% to 40%, the surface of the raw noodles became more smooth and flat (Figure 2A,B), whereas in cooked noodles, the hole size became larger (Figure 2C,D). For raw noodles, with the increase of GBP addition, the starch particles embedded into the gluten or gluten–fibre networks of noodles, resulting in the smooth surface of noodles. However, due to the lower concentration of gluten in GBP, the stability of the interaction of starch to the gluten network was decreased, which resulted in the higher cooking loss and larger size of holes in cooked noodles. Similar results were obtained from other studies [24,50].

### 3.9. In Vitro Starch Digestibility

**Figure 3:**
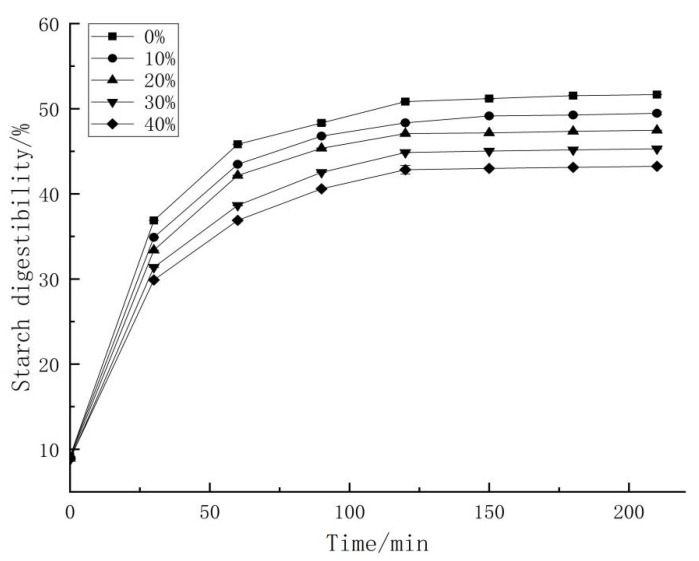
Starch digestibility of ginkgo biloba noodles.

To investigate the starch digestibility of GBN, in vitro enzymatic digestion was performed. As shown in Figure 3, starch digestibility of the GBN and reference white bread were increased with digestion time. During the first 60 min, the starch digestibility increased rapidly, and thereafter continued to gradually increase. This is consistent with a previous study by Tengnu Liu’s group [51]. Further, with the increase of GBP content, the digestibility of GBN was decreased. Meanwhile, starch digestion kinetics and estimated glycemic index (eGI) of GBN were also obtained, shown in Table 9. Equilibrium concentration of hydrolysis (C_∞_), hydrolysis kinetic constant (k) and the area under the hydrolysis curve (AUC) of digested GBN and white bread reference were obtained for the eGI value calculation. The eGI values of GBN decreased gradually with the increase of GBP addition. This was probably due to the low concentration of starch and the higher content of fibril in GBP than in the wheat flour, which decreased the starch digestibility and the eGI values of noodles [52]. Similar changes were observed by Meixia Fu and co-workers [53], when buckwheat was mixed with wheat flour and made into noodles.

## 4. Conclusions

The possibility of preparing noodles of acceptable quality from wheat flour supplemented with different levels of ginkgo biloba powder (GBP) was investigated in our study. We found that GBP would remarkably improve the quality of dough and fresh wet noodles. With increasing GBP addition, mixing tolerance index, water absorption, G′ and G′ of dough, and the extensibility, hardness, adhesiveness, springiness and chewiness of noodles were increased. However, the peak viscosity, trough viscosity, final viscosity, setback, breakdown, developing time, and stability of dough and the digestibility and eGI values of the noodles decreased. In addition, when the content of GBP was at 20%, the sensory scores of the noodles reached the highest value. For microstructure, GBP addition smoothed the surface of raw noodles but increased the hole size of the cooked noodles. In summary, when the GBP addition was less than 20%, GBP addition could increase the flavonoids content and had no significant negative impact on the acceptability of wheat noodles. This indicates that GBP (especially with the 20% addition) has the potential for promotion and application in the wheat noodle industry. Further studies are encouraged to determine the effect of the GBP particle size on the nutritional value and quality of the noodles.

## Figures and Tables

**Table 1:** Sensory evaluation standard of GBN.

Item	Full Score/Score	Marking Criterion
Color	10	Deep colour and bright 8–10; Light yellow and less bright 5–7; Light yellow 1–4
Appearance	10	Smooth and irregular shape 8–10; Less smooth and in shape 5–7; Rough and unshaped 1–4
Palatability	20	Suitable hardness 17–20; Harder or softer 12–16; Over-hard or soft 1–12
Toughness	25	Elastic 21–25; Poor elasticity 16–20; Worse elasticity 11–15; Worst elasticity 1–10
Viscosity	25	Non-sticky 21–25; Less sticky 16–20; Sticky hair 10–15; Very sticky 1–10
Smoothness	5	Smooth 5; Less smooth 3–4; Coarse 1–2
Flavor	5	Ginkgo flavor 5; Non-smell 3–4; Smell 1–2
Total score	100	Refined grade wheat flour products score ≥ 85; ordinary wheat flour products score ≥ 75

**Table 2:** Main chemical compositions of GBP/wheat flour mixtures.

Item	0%	10%	20%	30%	40%	100%
Moisture (%)	13.65 ± 0.44 ^a^	12.94 ± 0.09 ^b^	12.31 ± 0.04 ^c^	11.91 ± 0.06 ^d^	11.57 ± 0.12 ^d^	10.20 ± 0.11 ^e^
Protein (g/100 g)	17.41 ± 0.41 ^a^	16.55 ± 0.08 ^b^	16.39 ± 0.08 ^b^	15.67 ± 0.10 ^c^	15.34 ± 0.12 ^c^	13.75 ± 0.10 ^d^
Total sugar (mg/g)	656.61 ± 1.37 ^a^	654.32 ± 0.56 ^b^	651.95 ± 0.85 ^c^	649.24 ± 0.65 ^d^	646.62 ± 0.92 ^e^	639.23 ± 0.71 ^fc^
Starch (g/100 g)	63.59 ± 0.24 ^a^	60.73 ± 0.57 ^b^	57.30 ± 0.74 ^c^	54.68 ± 0.41 ^d^	52.20 ± 0.35 ^e^	34.83 ± 0.52 ^f^
Amylose (mg/g)	118.45 ± 2.00 ^f^	150.21 ± 7.22 ^e^	177.56 ± 1.77 ^d^	207.71 ± 2.31 ^c^	229.77 ± 0.55 ^b^	321.46 ± 3.33 ^a^
Gluten (g/100 g)	32.39 ± 0.49 ^a^	29.31 ± 0.36 ^b^	23.80 ± 0.27 ^c^	19.94 ± 0.43 ^d^	15.82 ± 0.34 ^e^	-
Fibre(mg/g)	0.87 ± 0.01 ^f^	0.93 ± 0.01 ^e^	1.03 ± 0.03 ^d^	1.11 ± 0.02 ^c^	1.17 ± 0.01 ^b^	1.56 ± 0.04 ^a^
Total flavonoids (mg/g)	-	0.29 ± 0.02 ^e^	0.51 ± 0.03 ^d^	0.78 ± 0.03 ^c^	0.97 ± 0.02 ^b^	2.00 ± 0.05 ^a^
Ginkgolic acid (mg/kg)	-	0.28 ± 0.03 ^e^	0.40 ± 0.02 ^d^	0.69 ± 0.04 ^c^	0.82 ± 0.04 ^b^	2.06 ± 0.09 ^a^

Note: means with different small letter superscripts within the same line are significantly different at *p* < 0.05. “-” indicates that the substance was not detected.

**Table 3:** Farinograph properties of GBP/wheat flour mixtures.

Sample	Water Absorption (%)	Dough Development Time (min)	Dough Stability Time (min)	Mixing Tolerance Index(FU)
0%	53.94 ± 0.39 ^e^	0.92 ± 0.06 ^b^	3.55 ± 0.05 ^a^	231.31 ± 15.06 ^d^
10%	54.9 ± 0.76 ^d^	1.03 ± 0.05 ^ab^	1.20 ± 0.14 ^b^	399.83 ± 14.69 ^c^
20%	55.57 ± 0.40 ^c^	1.10 ± 0.14 ^a^	1.07 ± 0.12 ^bc^	426.50 ± 22.48 ^c^
30%	57.08 ± 0.20 ^b^	1.17 ± 0.12 ^a^	0.93 ± 0.17 ^bc^	519.80 ± 12.25 ^b^
40%	58.00 ± 0.17 ^a^	1.20 ± 0.28 ^a^	0.80 ± 0.08 ^c^	579.80 ± 14.72 ^a^

Note: means with different small letter superscripts within the same column are significantly different at *p* < 0.05.

**Table 4:** Pasting properties of GBP/wheat flour mixtures.

Sample	Peak Viscosity (cP)	Trough Viscosity (cP)	Breakdown (cP)	Final Viscosity (cP)	Setback (cP)
0%	2621.00 ± 60.83 ^a^	1943.33 ± 69.81 ^a^	677.67 ± 11.90 ^a^	3091.67 ± 66.38 ^a^	1148.33 ± 46.02 ^a^
10%	1997.00 ± 40.42 ^b^	1404.33 ± 20.43 ^b^	592.67 ± 30.92 ^b^	2470.00 ± 46.61 ^b^	1065.67 ± 34.34 ^b^
20%	1762.05 ± 69.90 ^c^	1265.00 ± 44.86 ^c^	497.00 ± 25.39 ^c^	2226.67 ± 70.48 ^c^	961.67 ± 28.69 ^c^
30%	1414.67 ± 40.89 ^d^	1001.33 ± 31.37 ^d^	413.33 ± 10.21 ^d^	1835.00 ± 67.94 ^d^	833.67 ± 36.57 ^d^
40%	1268.67 ± 42.60 ^e^	894.67 ± 29.91 ^e^	374.00 ± 12.96 ^d^	1693.33 ± 49.09 ^e^	798.67 ± 19.60 ^d^

Note: means with different small letter superscripts within the same column are significantly different at *p* < 0.05.

**Table 5:** Effect of different levels of GBP on power-law model parameters in dough frequency scan tests.

Sample	G′ = K′ × W^n′^			G″ = K″ × W^n″^		
	K′ (Pa s^n′^)	n′	R^2^_1_	K″ (Pa s^n″^)	n″	R^2^_2_
0%	20,951.70 ± 137.45 ^e^	0.159 ± 0.002 ^c^	0.996	8376.21 ± 213.92 ^d^	0.227 ± 0.008 ^c^	0.977
10%	37,035.29 ± 186.07 ^d^	0.161 ± 0.002 ^b^	0.998	16,231.66 ± 278.27 ^c^	0.200 ± 0.005 ^c^	0.986
20%	55,210.75 ± 228.67 ^c^	0.153 ± 0.001 ^b^	0.998	22,527.66 ± 524.78 ^b^	0.210 ± 0.007 ^b^	0.977
30%	58,392.86 ± 445.39 ^b^	0.186 ± 0.002 ^a^	0.997	24,043.43 ± 634.59 ^a^	0.234 ± 0.008 ^b^	0.977
40%	66,692.16 ± 316.67 ^a^	0.187 ± 0.001 ^a^	0.999	23,472.34 ± 353.38 ^a^	0.251 ± 0.004 ^a^	0.994

Note: means with different small letter superscripts within the same column are significantly different at *p* < 0.05.

**Table 6:** Cooking properties of ginkgo biloba noodles.

Sample	Optimal Cooked Time (s)	Water Absorption (%)	Cooking Loss (%)
0%	336.66 ± 3.43 ^e^	4.10 ± 0.05 ^e^	6.11 ± 0.10 ^e^
10%	346.66 ± 4.22 ^d^	5.99 ± 0.13 ^d^	6.83 ± 0.10 ^d^
20%	383.33 ± 4.51 ^c^	9.09 ± 0.07 ^c^	7.51 ± 0.09 ^c^
30%	406.66 ± 2.45 ^b^	10.49 ± 0.48 ^b^	8.30 ± 0.06 ^b^
40%	433.33 ± 5.27 ^a^	11.20 ± 0.34 ^a^	11.43 ± 0.09 ^a^

Note: means with different small letter superscripts within the same column are significantly different at *p* < 0.05.

**Table 7:** Texture and tension properties of ginkgo biloba noodles.

Sample	Hardness (g)	Adhesiveness (g·s)	Springiness (g·s)	Chewiness (g·s)	Tensile Strength (g)	Elasticity Distance (mm)
0%	2588.13 ± 51.58 ^e^	64.73 ± 13.88 ^a^	88.01 ± 0.75 ^b^	1499.79 ± 25.24 ^d^	17.37 ± 0.32 ^e^	24.38 ± 0.33 ^a^
10%	2715.46 ± 6.04 ^d^	74.73 ± 7.88 ^a^	88.45 ± 1.25 ^b^	1586.82 ± 76.00 ^d^	20.30 ± 0.89 ^d^	26.75 ± 1.16 ^b^
20%	2923.57 ± 54.38 ^c^	81.54 ± 6.06 ^a^	90.44 ± 1.60 ^ab^	1728.82 ± 39.70 ^c^	24.42 ± 0.88 ^c^	28.08 ± 1.35 ^c^
30%	3095.09 ± 9.32 ^b^	99.42 ± 12.14 ^b^	91.54 ± 2.21 ^a^	1838.01 ± 51.93 ^b^	28.09 ± 0.16 ^b^	30.32 ± 2.12 ^d^
40%	3301.09 ± 45.36 ^a^	125.29 ± 1.45 ^c^	92.44 ± 1.78 ^a^	1981.41 ± 41.70 ^a^	32.26 ± 0.85 ^a^	35.08 ± 1.10 ^e^

Note: means with different small letter superscripts within the same column are significantly different at *p* < 0.05.

**Table 8:** Sensory properties of ginkgo biloba noodles.

Sample	Color	Appearance	Palatability	Toughness	Viscosity	Smoothness	Flavor	Total
0%	8.22 ± 0.19 ^b^	7.28 ± 0.13 ^bc^	18.02 ± 0.10 ^b^	20.14 ± 0.96 ^ab^	20.88 ± 0.53 ^ab^	4.38 ± 0.03 ^bc^	4.25 ± 0.09 ^c^	83.27 ± 2.09 ^bc^
10%	8.27 ± 0.24 ^b^	7.40 ± 0.13 ^ab^	18.42 ± 0.21 ^a^	20.54 ± 0.45 ^a^	20.95 ± 1.20 ^ab^	4.60 ± 0.15 ^a^	4.35 ± 0.15 ^bc^	84.37 ± 2.40 ^ab^
20%	8.57 ± 0.10 ^a^	7.50 ± 0.05 ^a^	18.08 ± 0.07 ^b^	20.93 ± 0.26 ^a^	21.76 ± 0.54 ^b^	4.55 ± 0.05 ^ab^	4.52 ± 0.15 ^ab^	86.33 ± 1.26 ^a^
30%	8.17 ± 0.08 ^b^	7.20 ± 0.05 ^bc^	17.87 ± 0.21 ^b^	20.22 ± 0.50 ^a^	19.80 ± 0.40 ^bc^	4.43 ± 0.13 ^ab^	4.60 ± 0.04 ^a^	81.94 ± 1.45 ^c^
40%	8.00 ± 0.05 ^b^	7.08 ± 0.15 ^c^	17.45 ± 0.18 ^c^	19.32 ± 0.22 ^b^	19.24 ± 0.38 ^c^	4.22 ± 0.08 ^c^	4.15 ± 0.05 ^c^	79.46 ± 1.11 ^d^

Note: means with different small letter superscripts within the same column are significantly different at *p* < 0.05.

**Table 9:** In vitro starch digestion kinetics and glycemic index determination of GBN.

Samples	Kinetics Parameters				
	C_∞_(%)	K × 10^−2^ (min^−1^)	R^2^	AUC	eGI
White Bread	59.22 ± 0.09 ^a^	4.24 ± 0.02 ^a^	0.9989	9068.42 ± 16.82 ^a^	94.40 ± 0.05 ^a^
0%	50.89 ± 0.12 ^b^	4.13 ± 0.01 ^b^	0.9982	7766.10 ± 22.74 ^b^	82.02 ± 0.47 ^b^
10%	48.82 ± 0.07 ^c^	4.01 ± 0.01 ^c^	0.9984	7421.16 ± 19.48 ^c^	78.74 ± 0.35 ^c^
20%	47.17 ± 0.62 ^d^	3.98 ± 0.03 ^c^	0.9993	7166.85 ± 17.31 ^d^	76.32 ± 0.31 ^d^
30%	44.85 ± 0.21 ^e^	3.75 ± 0.02 ^d^	0.9964	6752.25 ± 20.36 ^e^	72.38 ± 0.19 ^e^
40%	42.83 ± 0.14 ^f^	3.73 ± 0.04 ^d^	0.9972	6444.32 ± 19.35 ^f^	69.45 ± 0.62 ^f^

Note: means with different small letter superscripts within the same column are significantly different at *p* < 0.05.

## Data Availability

Research data are not shared.
